# Splenectomy as a Risk Factor for Graft Rejection Following Endothelial Transplantation: Retrospective Study

**DOI:** 10.2196/50106

**Published:** 2024-09-10

**Authors:** Paola Kammrath Betancor, Daniel Böhringer, Philip Maier, Thabo Lapp, Thomas Reinhard

**Affiliations:** 1 Eye Center, Medical Center, Faculty of Medicine, University of Freiburg Freiburg Germany; 2 Ophtha-Lab, Department of Ophthalmology, St. Franziskus Hospital Muenster Muenster Germany

**Keywords:** anterior chamber–associated immune deviation, ACAID, Descemet membrane endothelial keratoplasty, DMEK, splenectomy

## Abstract

**Background:**

Anterior chamber–associated immune deviation (ACAID) is an active immunotolerance mechanism, which is induced by placing antigen into the anterior eye chamber as long as a major surgical trauma is avoided. For this reason, ACAID may be a major contributor to the favorable immunologic outcomes in Descemet membrane endothelial keratoplasty (DMEK). Rodent models have demonstrated the importance of a functional spleen for the development of an ACAID.

**Objective:**

This study aimed to investigate whether splenectomy leads to increased rejection rates after DMEK in humans.

**Methods:**

A retrospective evaluation was conducted on the course following DMEK at the Eye Center, Medical Center, University of Freiburg, for patients with a self-reported history of splenectomy compared to patients without this condition. Potential study patients were contacted by mail. A questionnaire to self-report splenectomy and the time thereof was sent out. The medical records of all consenting patients at the Eye Center were reviewed for graft survival and immune reactions.

**Results:**

We asked 1818 patients after DMEK to report their history of splenectomy. A total of 1340 patients responded and were included in the study. Of these 1340 patients, 16 (1.2%) reported a history of splenectomy (ie, 26 DMEKs, with 10 patients being transplanted in both eyes and 6 patients being transplanted in 1 eye; median age at surgery 73.7, range 66.7-76.1 y). The remaining patients (1324 patients, ie, 1941 eyes) served as controls, with 1941 DMEKs (median age at surgery 71.5, range 64.1-77.2 y). Five (19%) out of the 26 eyes from the splenectomy group required a second transplant due to dislocation (n=2.8%), failure (n=2.8%), and rejection (n=1.4%). Kaplan-Meier analysis revealed no relevant difference compared with controls.

**Conclusions:**

Our results suggest that splenectomy has no major effect on the outcome following DMEK. Subsequent, ACAID may not be the main reason for the favorable immunological outcomes in DMEK, or the camero-splenic axis may be subordinate in humans. However, we only included 16 patients who underwent splenectomy, so it might be possible that we missed a minor effect.

## Introduction

Nowadays, corneal transplantation is one of the most common and successful forms of tissue transplantation worldwide, and in the vast majority of cases, it occurs without a human leukocyte antigen match [1]. Since not only can penetrating keratoplasty be performed but lamellar surgery techniques (such as Descemet membrane endothelial keratoplasty [DMEK]) are also available, the proportion of penetrating keratoplasties is decreasing while the number of DMEKs performed is increasing [2]. Approximately 2% to 7% of normal-risk patients after a DMEK and about 18% after a penetrating keratoplasty experience immunologic rejection [3-5]. At present, it remains unclear why there is less rejection in DMEK transplantation; consequently, a hypothesis arose—that the anterior chamber–associated immune deviation (ACAID) phenomenon may contribute significantly to the favorable immunologic outcomes in DMEK. As of today, there are practically no clinical data on this topic.

The fact that corneal transplantation can be effective was demonstrated by various surgeons and ophthalmologists in animal models as early as 1818 (on rabbits) [6]. The first successful corneal transplantation in humans was performed by Austrian surgeon Eduard Zirm [7] in 1906. In comparison, the first successful kidney transplantation was performed almost 50 years later, in 1953 [8]. Corneal transplants were much less likely to be rejected compared to organ transplants at very early stages after transplantation. The reason why the corneal tissue was not rejected directly led to many hypotheses about the immunology of the eye that allows this [9]. Considering these questions, clinicians as well as researchers began to investigate ACAID [10,11]. The immune system is able to prevent immune reactions against foreign antigens within the eye. In the rodent model, it has been found that this principle works only if the antigens are injected atraumatically into the eye [12]. The removal of the spleen prevents the development of ACAID in the rodent model, resulting in an increased rejection rate after corneal transplantation [13]. This is currently explained by the so-called camero-splenic axis. Antigen-presenting cells most likely derived from the iris and ciliary body take up antigens placed in the anterior chamber and migrate via the trabecular meshwork and collector veins through the blood into the spleen. Within the spleen, these antigen-presenting cells induce the differentiation of antigen-specific regulatory T cells, forming the cellular arm of ACAID [14]. However, there is currently no proof of this theory; human data on the presence of ACAID or the camero-splenic axis are lacking.

The primary objective of this retrospective study was to compare outcomes between human patients with and without splenectomy, specifically graft rejection and failure, following DMEK surgery. For this purpose, all reachable patients who underwent DMEK at the Eye Center, Medical Center, University of Freiburg, during the last 12 years (2734 eyes in total) were sent a questionnaire to self-report whether they underwent splenectomy and the time thereof. We herein investigate the potential role of ACAID in the excellent immunological prognosis of DMEK. This could provide initial evidence on the contribution of ACAID to the success rates of DMEK.

## Methods

### Study Design

This was a retrospective study evaluating the course following DMEK at the Eye Center, Medical Center, University of Freiburg, for patients with a self-reported history of splenectomy compared to patients without this condition.

### Data Collection

A total of 2734 DMEKs were performed between 2010 and 2022 at the Eye Centre, Medical Center, University of Freiburg. Patients who underwent DMEK during this period were eligible for inclusion. We were able to contact 1818 patients from this pool. They were sent a cover letter by mail, including patient information about the study, a consent form, and a questionnaire to self-report splenectomy and the time thereof. This questionnaire formed the basis of consent in our study. Patients who did not respond to the questionnaire or did not wish to participate in the study were excluded from the analysis.

The exposure of interest was a self-reported history of splenectomy, which was assessed through the questionnaire sent to patients. Data collected through the questionnaire included self-reported history of splenectomy and the time thereof.

The questionnaire was a simple 1-question survey asking patients to self-report whether they had undergone splenectomy and when. The questionnaire was developed by the research team and reviewed and approved by the ethics committee to ensure clarity and comprehensibility.

After submitting the questionnaire, patients cannot withdraw their participation in the research project, as we irreversibly anonymize their identity and destroy their questionnaire in accordance with data protection regulations once their information have been transferred for the statistical analysis. Therefore, individual study participants cannot be identified when publishing the study results.

Data collected from electronic health records included demographic information, indication for DMEK, and postoperative outcomes such as graft rejection and failure.

The medical records of all consenting patients were reviewed for graft survival and immune reactions. All patients are scheduled postoperatively for follow-up in our clinic, so it is highly likely that we have captured almost all immune reactions. These data were linked to the questionnaire responses to compare patients who underwent splenectomy to the remainder who served as controls.

DMEK surgery was executed per standards as previously described [3]. Briefly, a trephine was used to punch the grafts, which were stained with trypan blue 0.6 mg/mL. Subsequent to descemetorhexis and insertion of the graft into the anterior chamber, the graft was unfolded and centered. Next, complete air filling of the anterior chamber connected the graft to the posterior stroma. Postoperatively, patients were requested to remain supine for 3 days. Postoperative local therapy consisted of topical dexamethasone 5 times daily and tapered over 5 months to once daily, which was recommended for up to 24 months or longer. There was no evidence of any difference in postoperative medical aftercare between patients who under splenectomy and the controls.

Patients with postoperative epithelial defects were initially treated with dexpanthenol and ofloxacin ointment alternately every hour until epithelial closure, before the aforementioned treatment was started.

The main outcomes were graft rejection and failure.

Graft rejection was determined by reviewing medical records for signs of rejection, such as newly appearing endothelial precipitates on the graft [5]. Other clinical signs of graft rejection may include cells in the anterior chamber or otherwise unexplained corneal edema [15].

Corneal grafts that were not adherent and/or did not result in corneal transparency were classified as graft failures.

### Ethical Considerations

The study protocol was approved by the ethics committee of the Albert Ludwigs University of Freiburg (21-1472). Informed consent was obtained from all participants, and they had the ability to opt out of the study. Data were anonymized, and protective measures were in place to safeguard participant information. No compensation was provided to participants in this study.

### Data Presentation and Statistical Analysis

Descriptive statistics summarized baseline characteristics between the splenectomy and control groups. Median and IQR were reported for continuous variables, and percentages were reported for categorical variables. Differences were assessed using ANOVA for continuous data and Pearson chi-square tests for categorical outcomes.

Time-to-event analysis was performed using Kaplan-Meier curves depicting the risk of immune reactions, graft failure (as operationalized by regrafting), and rejection-free graft survival (a combination of the 2 aforementioned end points) between the splenectomy and control groups. Additionally, we also conducted another analysis by excluding early regrafting (during the first few postoperative days) due to technical failures. Groups were compared using the log-rank test. Multivariable Cox proportional hazard models were constructed to determine the independent association of splenectomy on outcomes after adjusting for potential confounding factors such as recipient age, sex, baseline diagnosis, and triple surgery (ie, cataract surgery in combination with DMEK). Hazard ratios with 95 CIs were reported.

Two-sided *P* values <.05 were considered statistically significant for all analyses, which were performed with R software (version 4.1.3; R Foundation for Statistical Computing) [16].

## Results

Of the 1818 patients contacted, 1311 (72.1%) responded by returning the questionnaire. Additionally, 11 (0.6%) patients responded by email, and 18 (1%) responded via telephone. This yielded a response rate of 73.7% (1340/1818). Among the 1340 respondents, 16 (1.2%) reported a history of splenectomy. Only 5 other patients reported back and did not want to be included in the study. The family members of 27 patients contacted us to inform us that the patient had died and that no information about splenectomy was available; thus, these patients were not included in the study.

Of the 1324 patients who did not undergo splenectomy, we performed DMEKs on both eyes among 617 patients, so a total of 1941 eyes could be included in the control group.

Of the patients who underwent splenectomy, some had received DMEKs on both eyes at our hospital, yielding a total of 26 DMEK procedures. Of the 16 patients who underwent splenectomy, 11 (69%) were able to recall the time of the surgery. The remaining 5 (31%) patients could not give information about the time of surgery. Only 2 patients (each having had a DMEK on only 1 eye) underwent the splenectomy after the DMEK (see Multimedia Appendix 1). Median age at transplantation was similar between the groups. However, there was a lower proportion of female patients (9/26, 35% vs 1129/1941, 58.2%) and a higher rate of triple surgery (20/26, 77% vs 1139/1941, 58.7%) in the splenectomy group.

After reviewing the medical records, we found that of the 26 DMEKs (16 patients) in the splenectomy group, 5 (19%) eyes required regrafting. Two of these repeated DMEKs were performed for technical reasons because of incomplete graft attachment. Only 1 patient presented the defined signs of endothelial graft rejection, and the remaining 2 patients showed unspecific late endothelial graft failure (see Table 1 and Multimedia Appendix 2).

**Table 1:** Summative evaluation of the Descemet membrane endothelial keratoplasties (DMEKs) of the 16 patients who underwent splenectomy.

Variable		Value
**Patients who underwent splenectomy, n**		16
DMEKs, n		26
**Repeat DMEKs (n=26 eyes), n (%)**		5 (19)
	Graft dislocation	2 (8)
	Graft failure	2 (8)
	Graft rejection	1 (4)

In the control group, graft failure occurred in 153 (7.9%) out of 1941 eyes, and rejections were observed in 26 (1.3%) eyes. We additionally compared the indication for DMEK between the patients who underwent splenectomy and the controls (see Multimedia Appendix 2). Of the 26 DMEKs in patients who underwent splenectomy, 24 (92%) had Fuchs endothelial dystrophy. Two patients had previously received penetrating keratoplasty and required a DMEK for graft failure. Precisely, these 2 cases required a second DMEK, both due to endothelial graft failure.

**Figure 1:**
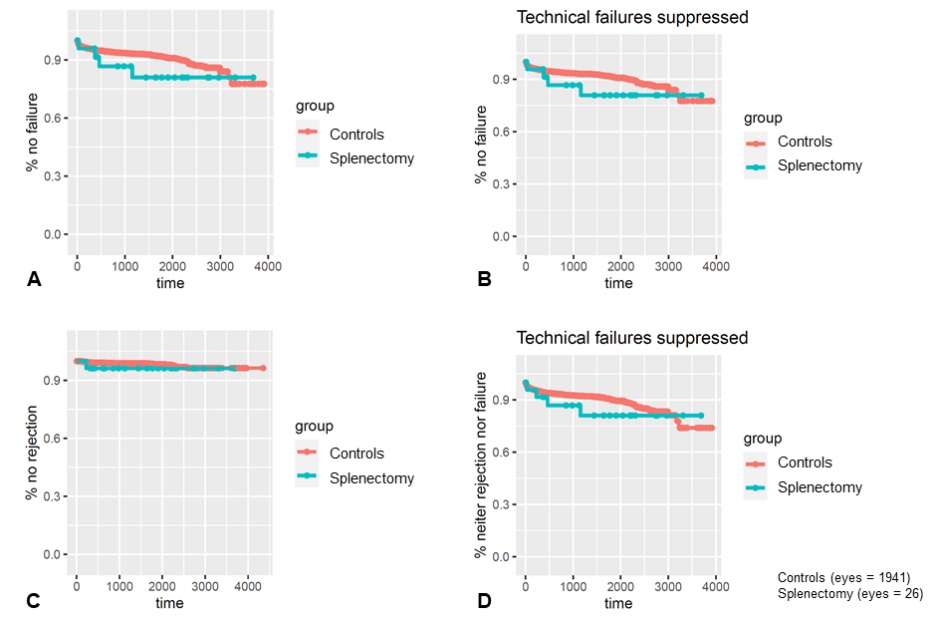
Survival analyses. (A) The percentage of patients who did not need a repeat keratoplasty shown over time in days. The patients who underwent splenectomy are presented in blue and the control group is shown in red. The splenectomy group includes the 2 cases of graft dislocation. (B) Without these technical failures, a slight trend of patients who underwent splenectomy toward the need of a second keratoplasty may be seen. The same plots are shown for (C) the rejection-free time and (D) without the technical failures. Included in these lower 2 curves is 1 rejection case that did not require repeat keratoplasty. Again, one could assume a trend here.

Kaplan-Meier analysis (Figure 1) and multivariable Cox proportional hazards models adjusting for age, sex, baseline diagnosis, and triple surgery revealed no statistically significant differences in the risk of regrafting, failure, rejection, or combined end points between groups (all *P*>.05).

Survival analyses comparing graft rejection rates, the need for repeat keratoplasties, and the combined results between groups are shown in Figure 1. The control group is considerably larger than the splenectomy group for obvious reasons. When counting the early repeat DMEKs from graft dislocation, the splenectomy group showed a trend toward a slightly worse performance compared to the controls (see Figure 1A). However, this is abrogated when counting only events that could theoretically be caused by immune reactions (see Figure 1B). In the comparison of the DMEK grafts without rejection reaction, almost no difference between the groups is discernable (see Figure 1C). Ignoring technical failures when regarding the combined results (see Figure 1D), a slight trend may be inferred toward the splenectomy group performing worse than the controls.

Specifically, the adjusted hazard ratio for the risk of graft failure comparing patients who underwent splenectomy to controls was 1.91 (95% CI 0.25-14.31; *P*=.53). Similar nonsignificant findings were observed for rejection and combined outcome models.

## Discussion

### Principal Findings

Our study was designed to investigate the effects of splenectomy on rejection after DMEK. Based on the data presented here, the spleen does not appear to have a major influence on the survival or rejection of DMEK grafts, so the significance of ACAID for human DMEK may be subordinate.

Animal models theorize that the spleen is essential in the induction of ACAID [13,14]; evidence to support this theory does not exist from clinical studies. This study is the first large-scale study to examine this in a retrospective clinical setting. We found no evidence to support these hypotheses.

It is still unclear whether ACAID and immune privilege for corneal transplantation established in animal models also applies to human keratoplasty and more specifically to DMEK. The low risk of allograft rejection after corneal endothelial transplantation is thought to be due to the ACAID phenomenon after the introduction of antigens into the anterior chamber [17]. As early as 1966, Streilein et al [18] demonstrated that external corneal procedures, such as keratoplasty, corneal cauterization, and corneal sutures, lead to inflammation that prevents the induction of ACAID. Yamada et al [19] examined the allogeneic response in the anterior chamber after the transplantation of corneal endothelial cells in a mouse model. Both intracameral injection of splenocytes and corneal endothelial cells induced ACAID with the suppression of the delayed hypersensitivity reaction. However, this could not be detected in inflamed eyes by cryoinjury, so the loss of the delayed hypersensitivity reaction does not seem to be regulated by ACAID.

Not all animal studies showed an effect of splenectomy on corneal graft survival. Bourne et al [20] performed corneal transplants in 19 rabbits each with and without splenectomy and found no significant difference in graft survival. In 2017, Vendomèle et al [11] summarized the evidence on ACAID and noted that several factors raised questions about the reliability and validity of studies using knockout mouse models. In particular, physiological relevance and transferability to humans must be considered critically.

To our knowledge, our study is the first with such a high number of patients who underwent both splenectomy and DMEK. Hos et al [21] followed a single case of a patient who underwent splenectomy for 4 years after DMEK. During this time, they noted no corneal graft rejection and assumed that the spleen was not necessary for graft acceptance.

Of our 26 DMEK transplants, 1 case developed graft rejection and 2 cases developed graft failure. In relation to the control group, this may suggest that splenectomy possibly does lead to a slight tendency of poorer graft acceptance. However, it has to be considered that the 2 graft failures are part of the high-risk group, because the indication for DMEK was the failure of a previously performed penetrating keratoplasty. Thus, from the data presented here, splenectomy does not appear to have a clear effect on the immunologic response to corneal transplantation. Furthermore, it should be noted that of all participating patients, only 16 patients who underwent splenectomy were included; thus, it is possible that we missed finding an influence on graft rejection after DMEK. The validity and reliability of the basic questionnaire was not formally tested. This is a limitation given the self-reported nature of the splenectomy history but is mitigated by the minimalistic (only 1 question) nature of this document. Additionally, we can conclude that undergoing splenectomy after DMEK does not have any influence either, since 2 of our patients showed such a course.

### Conclusion

As described previously, there is controversy about the relevance of the spleen in animal models investigating corneal graft rejection. The importance of the spleen in humans has not yet been investigated. Our results suggest that splenectomy does not substantially impact DMEK outcomes after accounting for potential confounding factors. However, given the limited sample size of patients who underwent splenectomy, a clinically meaningful effect cannot be definitively excluded. Nonetheless, ACAID may either not fully explain favorable immunologic outcomes following DMEK based on current evidence or the camero-splenic axis may be subordinate in humans.

## Appendix

Multimedia Appendix 1 Time course of the Descemet membrane endothelial keratoplasties (DMEKs) of the 16 patients who underwent splenectomy, including the indication for the DMEK.

Multimedia Appendix 2 Summarized data of the control and splenectomy groups.

## Abbreviations

ACAID: anterior chamber–associated immune deviation
DMEK: Descemet membrane endothelial keratoplasty
