# Supplementary material for: Splenectomy as a Risk Factor for Graft Rejection Following Endothelial Transplantation: Retrospective Study
Source: Interact J Med Res. 2024 Sep 10;13:e50106. doi: 10.2196/50106 (PMC11422749; doi:10.2196/50106)
Supplement: Multimedia Appendix 1 [file ijmr_v13i1e50106_app1.docx]

| **Splenectomized patients** | **Time of splenectomy** | **Eye** | **Indication for DMEK** | **Time of DMEK** | **Graft dislocation / failure / rejection** | **Re-DMEK** |
| --- | --- | --- | --- | --- | --- | --- |
| **1** | Date unknown | OD | FECD | 2015 | 0 | 0 |
|  |  | OS | FECD | 2016 | 0 | 0 |
| **2** | 1947 | OD | FECD | 2021 | 0 | 0 |
|  |  | OS | FECD | 2022 | 0 | 0 |
| **3** | 1978 | OD | FECD | 2016 | 0 | 0 |
|  |  | OS | FECD | 2015 | 0 | 0 |
| **4** | 2000 | OD | FECD | 2017 | 0 | 0 |
|  |  | OS | FECD | 2012 | 0 | 0 |
| **5** | Date unknown | OD | FECD | 2019 | 0 | 0 |
|  |  | OS | FECD | 2020 | 0 | 0 |
| **6** | 1974 | OD |  | / |  |  |
|  |  | OS | Penetrating graft failure | 2016 | **2019 graft failure** | 0 |
| **7** | 1985 | OD |  | / |  |  |
|  |  | OS | FECD | 2016 | 0 | 0 |
| **8** | 2017 | OD | FECD | 2013 | 0 | 0 |
|  |  | OS |  | / |  |  |
| **9** | 1960 | OD | FECD | 2020 | **2020 endothelial rejection** | 2021 |
|  |  | OS | FECD | 2020 | **2020 graft dislocation** | 2020 |
| **10** | 1997 | OD | FECD | 2014 | 0 | 0 |
|  |  | OS |  | / |  |  |
| **11** | Date unknown | OD | FECD | 2021 | 0 | 0 |
|  |  | OS | FECD | 2019 | 0 | 0 |
| **12** | 1945 | OD | FECD | 2018 | 0 | 0 |
|  |  | OS | FECD | 2017 | 0 | 0 |
| **13** | Date unknown | OD | Penetrating graft failure | 2020 | **2021 graft failure** | 2021 |
|  |  | OS |  | / |  |  |
| **14** | 2016 | OD |  | / |  |  |
|  |  | OS | FECD | 2014 | 0 |  |
| **15** | Date unknown | OD | FECD | 2018 | 0 | 0 |
|  |  | OS | FECD | 2021 | **2021 graft dislocation** | 2021 |
| **16** | 1969 | OD | FECD | 2021 | 0 | 0 |
|  |  | OS | FECD | 2018 | 0 | 0 |

FECD – Fuchs endothelial corneal dystrophy, OD oculus dexter, OS oculus sinister
