# Supplementary material for: Splenectomy as a Risk Factor for Graft Rejection Following Endothelial Transplantation: Retrospective Study
Source: Interact J Med Res. 2024 Sep 10;13:e50106. doi: 10.2196/50106 (PMC11422749; doi:10.2196/50106)
Supplement: Multimedia Appendix 2 [file ijmr_v13i1e50106_app2.docx]

|  | **Controls  (eyes = 1941)** | **Splenectomized patients  (eyes = 26)** | **Statistic Test** |
| --- | --- | --- | --- |
| **Age at the time of transplantation** | 64.1 / 71.5 / 77.2 | 66.7 / 73.7 / 76.1 | p=.736 |
| **Female** | 58% (1129) | 35% (9) | p=.016 |
| **Triple-DMEK**  **(Cataract surgery combined with DMEK)** | 59% (1139) | 77% (20) | p=.06 |
| **Indication for transplantation** |  |  |  |
| - **Fuchs endothelial dystrophy** | 92% (1791) | 92% (24) |  |
| - **Other corneal dystrophies** | 0% (9) | 0% (0) |  |
| - **Bullous keratopathy** | 6% (107) | 0% (0) |  |
| - **Graft failure** | 1% (17) | 8% (2) |  |
| - **Other** | 1% (11) | 0% (0) |  |
| **Postoperative** |  |  |  |
| - **Follow up (days)** | 322.0 / 861.0 / 1511.5 | 430.0 / 1543.5 / 2224.3 | p=.034 |
| - **Immune reaction** | 1% (26) | 4% (1) | p=.275 |
| - **Graft failure** | 8% (153) | 15 % (4) | p=.161 |
| - **Either graft failure or immune reaction** | 7% (134) | 15% (4) | p=.093 |
